# Maintenance of Skeletal Muscle Mass Does Not Guarantee Bench Press Performance: A Body-Composition Case Study

**DOI:** 10.7759/cureus.108557

**Published:** 2026-05-09

**Authors:** Khashayar Farzam

**Affiliations:** 1 Metabolic Medicine, True North Metabolic, Kitchener, CAN; 2 Health Sciences, McMaster University, Kitchener, CAN

**Keywords:** bench press, body fat, functional anatomy, powerlifting, primary care sports medicine, skeletal muscle mass, sports med, sports medicine, weightlifting, weight-loss

## Abstract

The bench press is one of the three powerlifting movements and is commonly viewed as a movement in which preservation of muscle mass should help preserve maximal performance. In practice, however, bench press strength output may also be influenced by body size, torso dimensions, and the mechanical conditions under which force is expressed. This case describes a decline in the one-rep max bench press during intentional weight loss despite relative maintenance of skeletal muscle mass on serial body-composition testing.

A 25-year-old male with a past medical history of prehypertension and dyslipidemia underwent serial body-composition testing over a 12-week period. At baseline, the body weight was 82.9 kg, and the one-rep max bench press was 172.5 kg (380 lb). At follow-up after a 12-week time period, the body weight had intentionally decreased to 76.0 kg, and the one-rep max bench press had decreased to 143 kg (315 lb). Body-composition analysis showed that the reduction in total body weight was driven predominantly by fat loss. Body fat mass decreased from 17.9 kg to 11.2 kg, the percentage of body fat decreased from 21.6% to 14.7%, and visceral fat area decreased from 78.5 cm^2^ to 48.3 cm^2^. In contrast, skeletal muscle mass changed only from 37.3 kg to 37.0 kg, lean body mass from 65.0 kg to 64.8 kg, and dry lean mass remained unchanged at 17.3 kg. Segmental upper-body lean mass also remained largely stable, with right arm lean mass changing from 4.13 kg to 4.07 kg, left arm lean mass from 3.99 kg to 4.01 kg, and trunk lean mass from 30.1 kg to 29.8 kg.

This case demonstrates that maintenance of skeletal muscle mass does not necessarily preserve bench press performance. In this patient, marked loss of fat mass and total body size occurred alongside a substantial decline in maximal bench press strength, despite only minimal change in measured skeletal muscle mass. This was during a time period in which strength training occurred. These findings suggest that non-muscle mass and body geometry may contribute meaningfully to bench press performance and may help explain reductions in bench press strength during weight loss, even when lean mass appears largely maintained.

## Introduction

The bench press is one of the three lifts contested in powerlifting and is widely considered a measurement of maximal upper-body strength. From a biomechanical standpoint, the lift depends primarily on force production by the pectoralis major, anterior deltoid, and triceps brachii, with additional contribution from the scapular stabilizers, latissimus dorsi, thoracic extensors, and trunk musculature. Electromyographic and kinematic studies of the bench press have shown that successful performance depends not only on the prime movers themselves, but also on bar path, setup, grip width, technique, and the lifter’s ability to maintain a stable position throughout the movement [1,2].

It is therefore reasonable that bench press performance is often discussed in relation to muscle mass. In simple terms, larger muscles are capable of producing greater force, and this is one reason why increases in muscle mass are often associated with improvements in maximal strength. At the same time, muscle size is only part of the picture. Muscular strength also depends on motor unit recruitment, rate coding, intramuscular and intermuscular coordination, technical efficiency, and familiarity with the movement pattern itself. Reviews of strength physiology have consistently shown that maximal force production reflects both muscular and neural determinants [3]. A lifter can therefore lose strength without a major change in muscle size, and conversely can improve performance without dramatic hypertrophy if technique and neuromuscular efficiency improve. That is why we can see powerlifters who are in lighter weight classes lifting heavier weights than those in heavier weight classes. 

Like in all of powerlifting, body size itself also plays an important role. In powerlifting settings, athletes often observe that body weight loss can impair the bench press disproportionately compared with other lifts. One likely explanation is that non-muscle mass contributes to a more favorable bench press position. A larger torso can shorten bar travel. Greater chest wall thickness and truncal girth can reduce the effective range of motion of the bench press. Increased overall body mass may also provide a broader, more stable base on the bench, making it easier to hold position, maintain tightness, and reproduce a reliable touch point. None of this implies that body fat generates force. Rather, it suggests that non-muscle tissue may improve the mechanical conditions under which pressing strength is expressed.

This point deserves more attention because reductions in bench press are often assumed to reflect muscle loss. In reality, a lifter may become lighter and mechanically less advantaged even if measured skeletal muscle mass changes little. This can lead to a decrease in strength performance despite maintaining muscle mass. This present case report illustrates an example of that.

Serial body-composition testing offered a way to explore this question more directly. The InBody 770 (InBody Co., Ltd, Seoul, South Korea) is a multifrequency segmental bioelectrical impedance device that estimates total body water, intracellular and extracellular water, lean body mass, dry lean mass, skeletal muscle mass, body fat mass, percent body fat, visceral fat area, and segmental distribution of lean and fat tissue with a high degree of accuracy. It also provides derived indices such as skeletal muscle index, phase angle, and the extracellular water to total body water (ECW/TBW) ratio. Prior validation work has shown that the InBody 770 has acceptable agreement with reference methods such as dual-energy X-ray absorptiometry (DXA) or multicomponent models when used for body-composition assessment, particularly for serial measurements and trend tracking [4,5]. In the present case, repeated InBody testing allowed comparison of whole-body skeletal muscle mass, upper-extremity lean mass, trunk lean mass, and regional fat distribution during a period in which bench press performance declined substantially.

## Case presentation

A 25-year-old male with a past medical history of prehypertension and dyslipidemia underwent serial body-composition assessment during a period of intentionally reduced body weight and reduced bench press performance. At baseline, the body weight was 82.9 kg, and the one-rep bench press was 172.5 kg (380 lb). At follow-up, body weight was 76.0 kg, and the one-rep max bench press was 143 kg (315 lb). This represented a reduction of 6.9 kg in body weight and 29.5 kg (65 lb) in maximal bench press.

The 25-year-old male patient was an experienced lifter with a background in competitive powerlifting. Following the first scan, they began a targeted weight loss program intended to reduce body fat mass and visceral fat. Throughout this time period, the 25-year-old male maintained a strength training routine including the bench press. There was a subjective and self-reported decrease in daily caloric intake.

Because the central question was whether the decline in bench press could be explained by the loss of muscle mass, attention was directed to skeletal muscle mass, lean body mass, trunk lean mass, and segmental upper-extremity lean mass. Two InBody 770 scans were available for review. The first and second scans were performed 12 weeks apart. Height was recorded at 167.5 cm on both assessments. The body-composition comparison was reviewed as a case study examining the discrepancy between bench press decline and preservation of measured lean mass, with particular emphasis on upper-body findings.

Results

The change in body composition and change in body weight occurred through intentional fat loss rather than by a measurable loss of skeletal muscle mass (Table 1). Total body weight dropped from 82.9 kg to 76.0 kg, a reduction of 6.9 kg. Body fat mass fell from 17.9 kg to 11.2 kg, accounting for 6.7 kg of the 6.9 kg weight decrease. The body fat percentage decreased from 21.6% to 14.7%, and the visceral fat area decreased from 78.5 cm^2^ to 48.3 cm^2^. Truncal fat mass showed the largest absolute reduction, falling from 10.3 kg to 6.1 kg. Fat mass in both arms and both legs also decreased.

**Table 1:** Selected body composition metrics at baseline and 12-week follow-up

Variable	Baseline	12-week follow-up	Absolute change
Weight, kg	82.9	76.0	-6.9
BMI, kg/m^2^	29.5	27.1	-2.4
Body fat mass, kg	17.9	11.2	-6.7
Percent body fat, %	21.6	14.7	-6.9
Visceral fat area, cm^2^	78.5	48.3	-30.2
Skeletal muscle mass, kg	37.3	37.0	-0.3
Lean body mass, kg	65.0	64.8	-0.2
Dry lean mass, kg	17.3	17.3	0.0
Skeletal muscle index, kg/m^2^	9.2	9.1	-0.1
Trunk lean mass, kg	30.1	29.8	-0.3
Right arm lean mass, kg	4.13	4.07	-0.06
Left arm lean mass, kg	3.99	4.01	+0.02
Right leg lean mass, kg	8.86	8.75	-0.11
Left leg lean mass, kg	8.74	8.78	+0.04
Trunk fat mass, kg	10.3	6.1	-4.2
Right arm fat mass, kg	0.9	0.3	-0.6
Left arm fat mass, kg	0.9	0.4	-0.5
Right leg fat mass, kg	2.3	1.5	-0.8
Left leg fat mass, kg	2.3	1.5	-0.8

In contrast to the changes in fat mass, the lean tissue measures were largely unchanged. Skeletal muscle mass changed from 37.3 kg to 37.0 kg, and lean body mass changed from 65.0 kg to 64.8 kg. Dry lean mass was 17.3 kg on both body composition scans. Skeletal muscle index changed only slightly from 9.2 kg/m^2^ to 9.1 kg/m^2^. Upper-body lean mass showed minimal variation between the scans. Right arm lean mass changed from 4.13 kg to 4.07 kg, and the left arm lean mass changed from 3.99 kg to 4.01 kg, and trunk lean mass changed from 30.1 kg to 29.8 kg. These changes were small in relation to the magnitude of the decline in the one rep max for the bench press.

Taken together, the scans showed a large reduction in total and regional fat mass with near-preservation of skeletal muscle mass and upper-body lean mass. The decrease in the bench press, therefore, occurred in the setting of substantial loss of non-muscle mass rather than any significant change in muscle mass.

## Discussion

The most notable feature of this case was the mismatch between the bench press performance and measured muscle mass. A substantial decline in the one rep max for the bench press would usually raise concern for loss of upper-body muscle mass, inadequate training in the gym, or some other reduction in force-producing capacity. The body-composition data we obtained did not support that theory. Whole-body skeletal muscle mass showed negligible change. Lean body mass changed very little as well. Segmental upper-extremity lean mass was essentially stable, even increasing in one arm. The scale and the bench press performance numbers changed much more than the lean mass measures did.

The largest measurable changes occurred in fat mass, especially in the trunk. That finding is important because the bench press is unusually sensitive to body dimensions [6,7]. When torso size decreases, the bar may have to travel farther down before reaching the chest. A reduced chest wall and abdominal profile can also alter the touch point and change the bottom position of the lift. In a movement performed from a fixed supine position, like the bench press, those changes can have major importance. The athlete may still have much of the same contractile tissue, but may no longer be bench pressing under the same mechanical conditions.

The present case supports the idea that bench press performance is influenced by more than skeletal muscle mass alone and that it is also influenced specifically by non-muscle mass. A lifter’s total body size, truncal thickness, and non-muscle tissue distribution may all affect how efficiently force is translated into a successful lift [6,7]. In that sense, body composition matters not just because of how much muscle is present, but because of how body mass is distributed and how that mass shapes leverage and stability.

This interpretation does not minimize the importance of muscular hypertrophy or neural factors in maximal strength output. Rather, it adds another layer of complexity for strength athletes. The athlete in this case became lighter and leaner, but not less muscular, according to serial body composition analysis. Yet the bench press strength performance still declined to a significant degree. For the bench press, reduced torso mass and reduced overall body size may have practical consequences that are not captured by muscle mass alone.

There are limitations to note. This is a single case and cannot establish definitive causative variables. Factors such as training structure, fatigue, bar path changes, psychological readiness, and recovery status were not formally measured. Even with those limitations, the pattern is clear enough to be clinically and practically relevant. The athlete lost a large amount of fat mass, retained most measured lean mass, and still experienced a major reduction in bench press performance.

## Conclusions

This case suggests that preservation of skeletal muscle mass does not necessarily preserve bench press performance. In a 25-year-old male powerlifting athlete, a significant decline in the one-rep max bench press occurred during a period of intentional weight reduction in which serial body-composition testing showed maintenance of skeletal muscle mass, lean body mass, and upper-extremity lean mass. The major change was loss of fat mass, particularly truncal fat mass. These findings support the view that non-muscle mass may contribute meaningfully to bench press mechanics through its effects on torso size, range of motion, and stability. This would be an optimal area of research to better determine the link between muscle mass and strength output.
